# Parental Experiences with Early Identification and Initial Care for their Child with Autism: Tailored Improvement Strategies

**DOI:** 10.1007/s10803-021-05226-y

**Published:** 2021-09-01

**Authors:** Michelle I. J. Snijder, Ilse P. C. Langerak, Shireen P. T. Kaijadoe, Marrit E. Buruma, Rianne Verschuur, Claudine Dietz, Jan K. Buitelaar, Iris J. Oosterling

**Affiliations:** 1 Karakter Child and Adolescent Psychiatry University Centre, Reinier Postlaan 12, Nijmegen, 6525 GC The Netherlands; 2 Department of Cognitive Neuroscience, Donders Institute for Brain, Cognition and Behaviour, Radboudumc, Nijmegen, The Netherlands; 3 Apanta GGZ, Veldhoven, The Netherlands; 4 INTER-PSY, Groningen, The Netherlands; 5 Dr Leo Kannerhuis, Arnhem, The Netherlands

**Keywords:** Autism spectrum disorder, Early detection, Preventive care, Parental experiences, Improvement strategies

## Abstract

Whereas it is well documented how parents experience the diagnostic process of their child with autism spectrum disorder (ASD), less is known about parental experiences with the course of the early identification process and first steps in receiving care for their child with ASD symptoms. This mixed-method study investigated these experiences as well as barriers and improvement strategies regarding early detection in the Netherlands. A parental survey (N = 45) showed that, on average, initial concerns started at 22 months. A focus group (N = 10) revealed multiple barriers and proposed strategies of improvement in three domains: “Knowledge and Expertise”, “Attention to Parental Needs” and “System and Organization”. Strategies to improve early identification will be discussed based on parental perspectives and professional perspectives.

**Supplementary Information:**

The online version contains supplementary material available at 10.1007/s10803-021-05226-y.

Over the last years, early identification of autism spectrum disorder (ASD) has been deemed a priority since scientific evidence indicates that timely identification and access to appropriate interventions can alter child development and have a positive effect on individual, family, and society level (Dawson et al., 2012; Estes et al., 2015; Fuller & Kaiser, 2019; Green et al., 2010; Horlin et al., 2014; Pickles et al., 2014). In the early identification of ASD and thus the start of early interventions, both parents and primary/preventive care professionals play a pivotal role. Early identification of ASD generally starts with parents expressing worries about their child’s development during the first 2 years of life (Landa, 2008; Zwaigenbaum et al., 2015), and in some cases, they even express their concerns before their child’s first birthday. Unfortunately, large gaps varying between 1.5 and 3.5 years are common between initial concerns and receiving a clinical assessment and ASD diagnosis (Crais et al., 2020; Crane et al., 2016; Zuckerman et al., 2015). Due to this delay, parents often describe the process of obtaining an ASD diagnosis for their child as stressful (Crane et al., 2016). One the one hand, parents are (in some cases) the first ones to express concerns regarding the child’s development. On the other hand, there are cases where first line healthcare professionals such as preventive care physicians (PCPs) express initial concerns. For PCPs, it can be challenging to discuss these concerns with parents. For example, when parents do not seem worried about the possibility of their child having ASD, PCPs find it difficult to motivate parents for referral to more specialized facilities. Also, according to PCP’s, parents often try to avoid a diagnosis for their child at such a young age (Snijder et al., 2021). All in all, sometimes it appears to be difficult for parents and PCPs to understand and meet each other’s point of view. To reduce the delay and improve early identification of ASD it is highly important that the experiences and perspectives of parents in this process are well understood.

Recognizing this importance, several studies have explored parents’ perspectives on the process of their child being diagnosed with ASD. Parents often report dissatisfaction with this process, due to the significant delay between initial concerns and the actual diagnosis (Chamak et al., 2011; Chao et al., 2017; Crane et al., 2016; Ho et al., 2014). Furthermore, these studies primarily focus on parental experiences and satisfaction with the *diagnostic process* itself and find that parents regularly experience the process as a stressful event, accompanied by feelings of shock, despair, depression, frustration, grief, worry and other negative emotions (Boshoff et al., 2018). Contributing to the stress of parents are an initial incorrect diagnose for their child, dissimilar advice, alternative explanations, a long and complex diagnostic process, limited support following diagnostic evaluation and frustrating paths to access appropriate healthcare services (Boshoff et al., 2018; Carlsson et al., 2016; Crais et al., 2020; Reed & Osborne, 2012). On the other hand, many parents eventually report a sense of relief and feeling validated by the diagnosis of their child (Boshoff et al., 2018). Although the experiences of parents regarding the diagnostic process of their child are relatively well documented, less is known about parental experiences during the first phase of early identification and discussing initial concerns with PCPs.

The few recent studies that looked into parental experiences during the phase of early identification suggest that parents find it difficult to discuss their initial concerns due to their concerns being dismissed or professionals adapting a ‘wait and see’ approach, instead of validating parental concerns (Johnson et al., 2020; Locke et al, 2020). Other barriers experienced by parents during early identification were the lack of using ASD-specific screening tools by PCPs and a lack of knowledge regarding appearance of ASD in girls and underrepresented populations (Crais et al., 2020; Locke et al., 2020). These previous studies have all been conducted in the United States, where the American Academy of Pediatrics (AAP) guideline recommends universal screening (i.e., all children at 18 and 24 months are screened for ASD; American Academy of Pediatrics, 2020). However, less is known about parents’ perspectives with the early identification process in countries where another approach to screening is being followed (i.e., screening in high risk groups). The current study is one of few studies conducted outside of the US to take parents perspectives into account in a country where there is no universal screening for ASD (i.e., parents are likely to be the first ones to identify initial concerns).

Therefore, the current study will explore parental perspectives with regard to the early identification of ASD in the Netherlands. The Dutch national guideline recommends that children should be specifically screened for ASD when one or more red flags for ASD are identified during general health surveillance at the well-baby clinic (van Berckelaer-Onnes et al., 2015). Well-baby clinics are a form of preventive healthcare where physicians and nurses have systematic contact with young children and their families during the first 4 years of life, mostly for routine vaccinations and developmental surveillance. In the current study, three specific questions will be addressed: (1) what were parents’ initial concerns regarding their child’s development and when were they expressed; (2) what barriers did parents experience in the early identification process of their child with ASD and (3) according to parents, which strategies are needed to improve early identification and access to healthcare of young children with ASD? This study is linked to a study of perspectives and experiences of preventive care physicians (as opposed to parents) regarding early identification of ASD, which covers the same research questions though from a different angle (Snijder et al., 2021). Hence, in the discussion section, results of the current study will be integrated with the PCPs viewpoints on the same topic, as investigated by a previous study of our group (Snijder et al., 2021).

## Methods

### Study Design

The current study engaged a two-stepped, mixed-method design. The first step involved an online survey (N = 45) targeting the process from initial concerns to enrollment in specialized mental healthcare services for diagnosis and intervention. The second step consisted of an additional, exploratory focus group (N = 10) to gain more in-depth insight into shared impressions of our target group (Rubin & Rubin, 1995). Ethical approval was obtained from local institutional review boards (CMO Radboud University Medical Centre and Karakter Child and Adolescent Psychiatry University Centre and Leo Kannerhuis). For the qualitative part of the study, the authors followed the Consolidated Criteria for Reporting Qualitative Research (COREQ; Tong et al., 2007) (see Supplementary Table 1).

### Inclusion, Recruitment and Procedures

#### Online Survey

An estimated 300 parents of children diagnosed with ASD at age six or younger diagnosed (between 2016 and 2018) were approached and invited to participate in reflecting back on their experiences pre-diagnosis through an online survey. The only exclusion criterion was insufficient mastery of the Dutch language to complete an online survey. Recruitment efforts occurred via a national expert network of clinicians and researchers involved in early autism. Experts from leading mental healthcare organizations across the Netherlands, who are participating in this network, were encouraged to approach parents from their caseloads. Eventually, a total of six mental healthcare organizations spread across the country (central, northern and eastern region of the Netherlands) recruited potential participants. The potential participants received an e-mail invitation via the organization they were linked to (the research team had no direct access to most of the potential participants) containing full information on the nature of the study and including a link to the online survey. The online survey could only be completed when parents had given informed consent. The survey was fully anonymous with no personal identifying information. Because of this, non-responders could not be identified and no reminders could be sent by the research team, causing a low response rate (estimated < 15%). In order to gain more in-depth insight in the experiences of parents with the process of early identification, the second step was hosting a focus group.

#### Focus Group

Parents of children diagnosed with ASD at 6 years or younger were asked to participate. Again, the only exclusion criterion was insufficient mastery of the Dutch language to participate in a group discussion. Recruitment efforts occurred through contacting healthcare professionals at two specialized mental healthcare centers in the eastern region of the Netherlands. These healthcare professionals were encouraged to approach suitable parents from their caseloads. Furthermore, recruitment efforts consisted of posting flyers in the offices of general practitioners and in well-baby clinics and by posting flyers on social media. Ten parents who were interested to participate in a focus group discussion contacted the research team by e-mail. Then, after receiving full information of the study by phone and signing the informed consent form, all parents were invited to participate in the focus group discussion. Recruitment stopped after the maximum number of participants (N = 10) was reached. There were no drop-outs. The possibility of hosting a second focus group was kept open, depending on the outcomes, but was not held for reasons mentioned below (see Data Analysis). As a sign of appreciation participants received a €20 gift card.

### Participants

#### Online Survey

Overall, 39 birthmothers and 6 birthfathers (total N = 45) completed the online survey. Originally, 46 parents completed the online survey but one parent was excluded from data analysis since he or she did not meet the inclusion criteria (other diagnosis, no ASD).

All participants reported in name of both parents. In total, 39 mothers and 43 fathers were native Dutch individuals. Three mothers were of other European nationality (6.6%), one was Mongolian (2.2%), one was Turkish (2.2%) and one was from the Dutch Antilles (2.2%). One father had the Colombian nationality (2.3%). Information about one father was missing. Participants reported on the early identification process and diagnostic assessment trajectory of 45 children. The majority of these children was male (82%). All 45 children had the Dutch nationality, three (6.7%) had a second nationality (British, German or Italian). All children were diagnosed with ASD. In total 11.11% of the children had a comorbid disorder: ADHD (4.4%) intellectual disability (4.4%) and selective mutism (2.2%). See Table 1 for more details on family and child characteristics.

**Table 1:** Family characteristics of the online survey

	Father (N = 44)	Mother (N = 45)	Child (N = 45)
Mean age (year; months), and range	37; 9, (26.0–60.0)	35; 10, (24.6–45.5)	5; 9 (3.0–8.4)
Level of parental education			
Primary/junior vocational	2.3%	2.2%	–
Secondary^a^	63.6%	53.3%	–
Higher professional/university	34.1%	44.4%	–
Primary diagnosis			
None	56.8%	62.2%	–
ASD	11.4%	2.2%	100%
Suspected ASD^b^	2.3%	–	–
ADHD	9.1%	2.2%	–
Anxiety	2.3%	2.2%	–
Depression	2.3%		–
Stress/burnout	6.8%	6.7%	–
Comorbidity	9.1%	24.4%	11% ^c^

^a^Junior general secondary, senior secondary vocational, senior general secondary and pre-university

^b^Diagnosis not confirmed

^c^All children were diagnosed with ASD; 11% of them had a comorbid disorder

#### Focus Group

In total, ten parents participated in the focus group discussion. All participants were birth-parents (nine mothers and one father) and were native Dutch individuals. The age varied between 37 and 52 years old, with an average age of 43.5 (*SD* = 5.49) years. Their average education was of higher professional/university level. Parents reported on twelve children in the focus group: ten boys and two girls. Most parents shared their experiences with one child diagnosed with ASD. Two parents shared experiences with two children, one of which concerned twins. Children received an ASD diagnosis between the ages of three and six. Eight children were recently diagnosed (average 1; 3 years ago), four were diagnosed longer ago (respectively 6, 8, 9 and 11 years ago).

### Measures and Data Collection

#### Online Survey

Data collection ran from February 2019 to August 2019. The survey was based on a literature search (Crane et al., 2016) and was designed to fit the population and research questions. The online survey was tested by the authors and pilot tested by a parent of a young child with ASD, matching inclusion criteria. Few adaptations were made after pilot testing. The final version of the survey consisted of demographic questions and six topics. With the aim of the current study, only the first two topics of the survey (initial worries and searching for help) were included in the data analysis. Other topics included: receiving a diagnosis for their child, treatment, child education and overall satisfaction with the healthcare process. The full survey can be found in the Supplementary Materials.

#### Focus Group

The focus group was organized in November 2019 and was conducted by a research team consisting of one PhD candidate who was trained in conducting qualitative research (MS as hostess), one child and adolescent psychiatry resident (IL as note taker) and one researcher experienced in qualitative research (SK as facilitator). All three were women. Every effort was made into creating a safe environment for all participants to ensure they felt secure in sharing their experience (Tausch & Menold., 2016). Upon receiving verbal consent, the focus group was audio recorded. The note taker and facilitator made a detailed record of nonverbal cues of participants. The discussion lasted approximately two hours and was held on a weekday evening at the clinic. During the focus group, three questions were posed: “When did your initial worries start concerning ASD and the development of your child”? “What was this like: discussing these concerns with others (family, professionals etc.)”? and “What could have been done better in the process from initial concerns to the start of diagnostic assessment”?

### Data Analysis

#### Online Survey

Respondents were asked what their initial concerns were regarding their child’s development. Percentages for different types of initial concerns were calculated. Furthermore, descriptive statistics were used to calculate the average time difference between a child’s age at initial concerns and at diagnosis. Also, the average severity of first concerns as rated on a 5-point Likert scale (with 1 being *not worried* and 5 being *very worried*) where calculated for mothers, fathers and first line healthcare workers as indicated by the respondent. A paired samples t-test was conducted to compare concerns between parents and professionals. The respondent that completed the questionnaire reported the severity of concerns for themselves, the other parent, and the first line healthcare workers (preventive care physicians and general practitioners), hence the choice to conduct a paired samples t-test.

#### Focus Group

The audio recording was transcribed verbatim and de-identified by a research assistant with a psychology background. Accuracy was checked afterwards by all researchers present at the focus group. The transcripts were not returned to participants for corrections. Furthermore, participants were not involved in the analyses of the data. Transcripts were coded and analyzed using an iterative process of group coding and thematic analysis (Braun & Clarke, 2006). Participants’ quotes were hereby the starting point in constructing themes and categories. After familiarizing with the data by reading and re-reading, coding was first roughly done by hand, by the first and second author, followed by comparing codes and discussing differences to derive inter-coder reliability. Codes were entered in Atlas Ti Qualitative Analysis Software, version 8. Through discussion and agreement over discrepancies with the first, second and third author, a consensus over the codes was reached. Similar segments were reassembled into categories. Then categories were compared and put together to form themes. No themes were identified in advance. Finally, since traditional data saturation cannot occur when only one focus group is held, the first author verified the extensiveness of the emerged themes by comparing the focus group results with answers given by participants during the online survey. Based on these open answers, no additional themes were identified and therefore it was decided not to host a second focus group since thematic saturation was reached.

## Results

### Initial Concerns

**Fig. 1:**
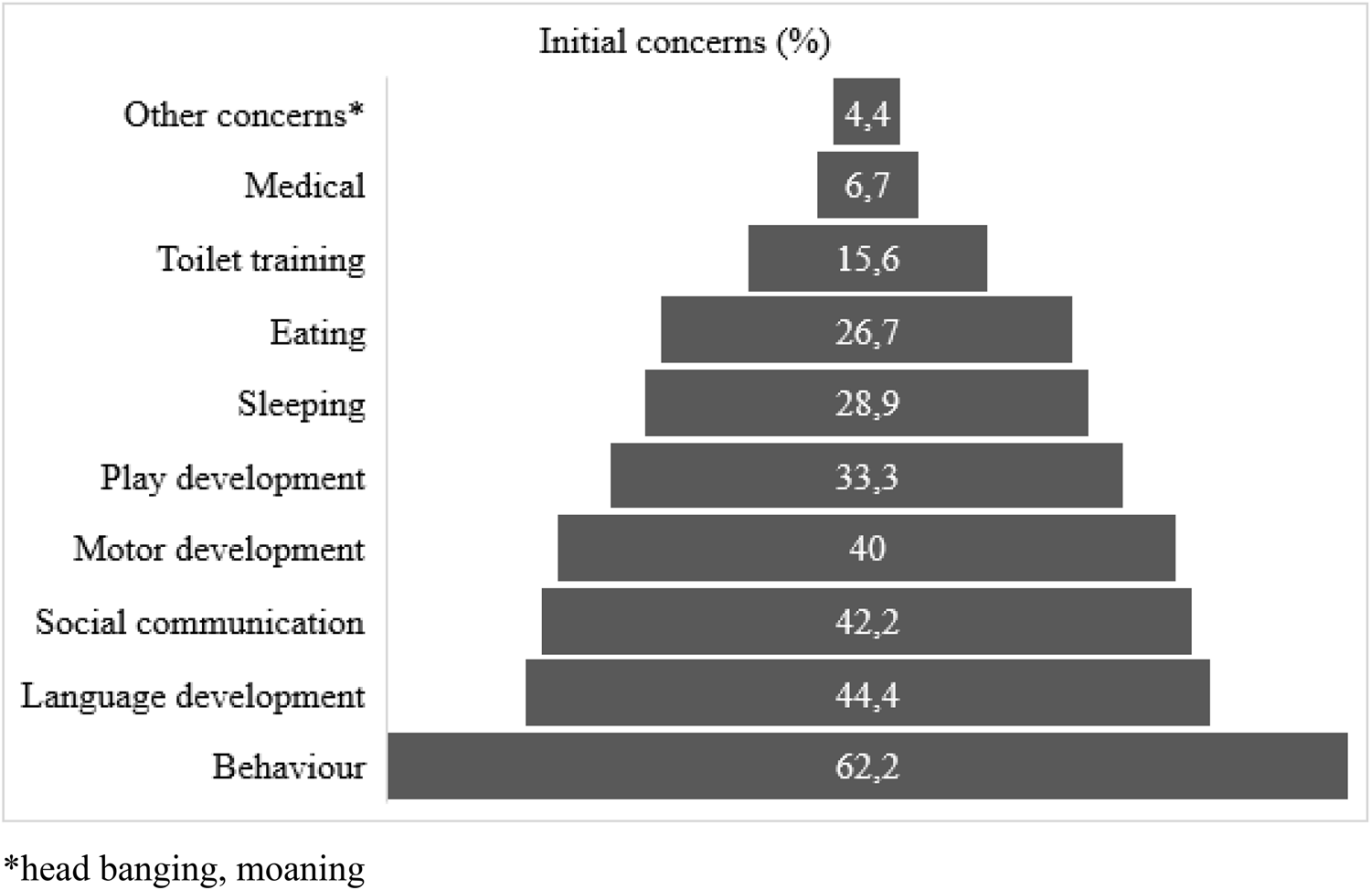
Overview of initial concerns, as reported by parents

This section covers the results extracted from the survey data. Initial concerns by parents were expressed when the child had an average age of 22 months (range 0–48, *SD* = 12.96). Upon receiving an ASD diagnosis, children had an average age of 48 months (range 24–76, *SD* = 15.35), meaning an average gap of 26 months between first concerns and an ASD diagnosis. The kind of initial concerns and alarm signals, as reported by parents, are entailed in Fig. 1. Behavior concerns (for example tantrums, anxious behavior and/or self-determined behavior) were most frequently reported (62.2%), followed by concerns related to the child’s language development (44.4%). Overall, parents reported that they had visited a wide range (0–6) of different healthcare professionals before receiving an ASD diagnosis for their child. On average, parents visited two or three different healthcare professionals (for example a speech therapist, physiotherapist, pediatrician, child psychologist) before their child received an ASD diagnosis. Lastly, the severity of initial concerns regarding child development (as perceived by the respondent) amongst mothers, fathers and first line healthcare workers (preventive care physicians and general practitioners) was calculated. Overall, parents (M = 3.64, SD = 0.89) seemed to report more severe initial concerns than first line healthcare workers (M = 2.55, SD = 1.10, *t*(41), *p* < 0.001). Specifically, there was a significant difference in the severity of concern by mothers (M = 3.89, SD = 0.98) compared to the initial concern of fathers (M = 3.27, SD = 1.03), *t*(42), *p* = 0.001.

When asked how satisfied parents were with the trajectory from first concerns to receiving a diagnosis, parents reported a varied range of satisfaction (1–5, with 1 being *not satisfied* and 5 being *very satisfied*). Generally, the majority of parents reported to be either satisfied or very satisfied with the process from first concerns to diagnosis (55.6%). The average satisfactory score was 3.49 (*SD* = 1.46). Overall, this score implies that parents are rather satisfied with the early identification process of ASD, but that there is still room for improvement. The experiences of parents with early identification and initial care are further explored in the focus group, along with proposed improvement strategies.

### Barriers and Improvement Strategies in Early Identification of ASD

This section covers the themes extracted from the focus group data. Based on their experiences, parents shared their struggles and proposed ideas for improvement, further referred to as ‘barriers and improvement strategies’ in the process of early identification of ASD. A description of three themes that were constructed from the focus group data is given below. Additional quotes are presented in Table 2. Although the emphasis was on the early identification process of ASD in primary and preventive care, parents additionally shared their experiences of the diagnostic process and treatment. As we consider this information beyond the scope of the current study but as additional interest, these findings are presented in the Supplementary Materials.

**Table 2:** Themes and categories representing barriers, accompanied by illustrative quotes

1. Knowledge and Expertise	‘What I would like to specify is that there is not enough knowledge at the general practitioners and at the well-baby clinics regarding ASD signals and young children’. Participant 6 ‘First they referred us to a child psychologist. We had an intake there and three days later, they called us. They couldn’t begin anything with such a young child, showing this kind of behavior. So, they couldn’t help us’. Participant 5 ‘If the healthcare professionals just would notice what early signals are…’ Participant 1
2. Attention to parental needs	
2a. Acknowledgement of parental concerns	‘When my child was 1.5 years old, I recognized the signs again [older sib already diagnosed with ASD]. They didn’t have concerns in preschool but still consulted a behavioral psychologist. She did not recognize the red flags either, nor the general practitioner, nor the preventive care professional at the well-baby clinic. Then I thought: it must be me, I am projecting things’. Participant 6 ‘Well, they literally see you as an overprotective mother. Or in my case, someone who brings her work home and project on her own child. Yes, I’ve heard it all’. Participant 5
2b. Risk of social isolation, prejudice and stigma	‘They [family and friends] often told me “you spoil him too much, you are always carrying him” and I would think: yes but if I don’t he will not stop crying’. Participant 2 ‘We do not have social lives (…). And people move on because ‘there are always “issues” with us.’ Participant 8
3. System and organization	
3a. Inflexibility of the system	‘We always had good contact with the staff members at preschool. They alwas said; “there is something special about [Name]”, but they always looked at what[Name] needed. If I hear other’s stories, I realize I have been lucky. That we had a preschool that always acted according to [Name]’s needs. If he for example needed one-on-one guidance, they always offered it’. Participant 4 ‘We persued a diagnosis since, otherwise, we wouldn’t get any mony from the muncipality to pay for care. They were born premature and that wasn’t enough to meet criteria. You are being pushed into the direction [of diagnostic assessment] just because you have to’. Participant 8
3b Waiting lists	‘Everybody says: ‘let’s wait, maybe things will improve in time’. Then follows a waitlist: we have to wait six months until someone is available. Then, another waitlist, and before you know it a full year has passed’. Participant 1 ‘I called them [specialized healthcare] and—maybe I don’t have enough patience and I don’t know how the systems works—but I called them and she said: you have the know, we have to screen the file first, that takes time’. Yes, but how long does it have to take? For a mother, it takes very, very long.’ Participant 9
3c. Fragmentation of care	‘We have had meetings with ten or twelve people. We, two parents, the rest of them all therapists and doctors’. Participant 8 ‘City X, I hear has very good care. Then they jokingly say: “you should have moved to city X’. Participant 3 ‘Much depends on the muncipality, where you live’. Participant 10

#### Knowledge and Expertise

Knowledge and Expertise was the first theme constructed from the data. Parents expressed their concerns about the lack of knowledge and expertise regarding early signs of ASD when consulting preventive care professionals and general practitioners. Some parents felt that preventive care physicians and nurses only focused on the average developmental milestones (e.g., growth, speech, motor skills) instead of acknowledging parents’ concerns of ASD and did not identify the behavioral red flags of ASD. However, two parents expressed to have positive experience regarding early identification of ASD at the general practitioner’s office, where a specialized healthcare professional (i.e., a psychologist or a child psychiatrist) was available to provide consultation and address their concerns. Therefore, as an improvement strategy, parents recommended additional training in recognizing the early symptoms of ASD in infancy and toddlerhood for primary and preventive care professionals.

#### Attention to Parental Needs

Attention to parental needs was the second theme and was divided into the barriers regarding: (a) acknowledgement of parental concerns, and (b) risk of social isolation, prejudice and stigma.

##### Acknowledgement of Parental Concerns

Parents experienced their concerns to be easily dismissed and marginalized by preventive care professionals and general practitioners. When parents discussed their initial worries of ASD, they mentioned that preventive care professionals (physicians and nurses) and general practitioners often would first try to comfort the parent, ensuring them that there was “nothing wrong” with their child, instead of asking more questions in order to clarify parental concerns. However, marginalizing and dismissing concerns did not lead to parents feeling relieved. Instead, parents felt insecure, thinking: ‘if there is nothing wrong with my child, then there must be something wrong with *me* that I feel this way.’ Parents emphasized that it is of great importance that they feel heard when initially contacting preventive and primary professionals about their concerns. Now, parents sometimes experience that they are being dismissed as an overly concerned parent. Parents value a healthcare professional that supports the child and their family, is easily available and is willing to go the extra mile.

##### Risk of Social Isolation and Prejudice

Many parents described a risk of social isolation: they had to start working part-time, or even resign entirely, because their child could not fully function or thrive when attended in daycare, by other family members (i.e., grandparents), or by babysitters. Some parents mentioned that they feel judged by their family, friends and healthcare professionals: as if their child’s divergent behavior is caused by their parenting style. These experiences cause parents to feel shame, guilt and frustration. Improvement strategies on how to target the risk of social isolation were not discussed amongst parents, since they almost all struggled with it. According to parents, an improvement strategy on how to reduce prejudice amongst first line healthcare workers was to increase their knowledge about ASD in young children, so that a better distinction can be made between child behavior, ASD symptoms and parenting styles.

#### System and Organization

The third and last theme surfaced from the data was Systems and Organization, divided into: (a) inflexibility of the system, (b) waiting lists, (c) fragmentation of care.

##### Inflexibility of the System

The majority of parents described various struggles due to the inflexibility of the (healthcare) system. Parents describe having to push and complain to their preventive care physician or general practitioner in order to get a referral to the specialized mental healthcare for their young child at risk of ASD. Additionally, parents experienced inflexibility of the system regarding daycare and schooling facilities. Parents mentioned that they were obligated to switch to a different daycare and/or schooling facility when their child did not “fit in”. Instead, parents wished that their child would have received additional and adequate guidance at regular daycare and schools. Parents stated the need for improved, customized care, since parents now felt that they were forced to conform and adapt to the healthcare system. Two parents during the focus group mentioned an opposite experience: they felt that their regular daycare was able to provide adequate guidance and support.

##### Waiting Lists

Next, parents were often confronted with long waiting lists, both for behavioral experts connected to daycare facilities and intakes at specialized mental healthcare centers. According to parents, waiting lists caused unnecessary delays and feelings of annoyance and impatience (“being sent from pillar to post”) and prolonged suffering due to worries and insecurities of parents.

##### Fragmentation of Care

Parents felt that the healthcare system is divided into “isolated little islands” where, for example: (a) a general practitioner or preventive care physician refers the child and its family, (b) a psychologist and/or psychiatrist is involved during the intake process (c) one or more other professional(s) provide(s) the diagnosis, (d) sometimes another professional offers therapy and treatment, etcetera, (e) another healthcare organization is involved in the family for proving care to a sibling, (f) a professional from the municipality is involved etcetera. Due to this fragmentation, within and between healthcare organizations, parents found it difficult to find the right person at the right time. This fragmentation, as mentioned by parents, causes unnecessary delays and prolonged periods of stress for the child and its family. Parents wished for a (lifelong) case manager: someone who keeps an overview of all the different parties involved in the care of the child at risk of ASD and supports parents in navigating the healthcare system. Enhancing communication and collaboration between all parties would, for an example, be a job for the proposed case manager. Parents wished for this case manager to also provide emotional support for parents. Furthermore, parents mentioned the importance of a closer collaboration between first line healthcare and specialized mental healthcare. As an example, two parents recalled their positive experience with a child psychiatrist offering consultation at the general practitioner’s office, providing a quick referral to the specialized mental healthcare. Lastly, parents mentioned that the availability and quality amongst care providers can differ a lot and is often highly depending on the family’s residential area. Thus, parents stated the need for quality checks on (ASD) healthcare providers, due to this variety in quality and availability.

## Discussion

This mixed-method study investigated the experiences of parents regarding the first steps (before diagnosis) in the process of getting help when they themselves and/or their professional signaled developmental problems and/or suspicions of autism spectrum disorder (ASD). From parents’ perspective, barriers in the early identification of young children with ASD, as well as potential improvements were studied. During the first phase of the study, an online survey was completed by 45 parents. During the second phase, an additional focus group was hosted with ten parents. Generally (as becomes apparent from questionnaire data), findings seem to indicate that this group of parents experience more severe concerns regarding ASD and their child’s development than first line healthcare workers (preventive care physicians and general practitioners), and they expressed to be fairly satisfied with the early identification process. Parental initial concerns include a variety of worries. Additionally (as discussed in the focus group), parents revealed several barriers in the process of getting access to appropriate healthcare and suggested improvement strategies in the early identification ASD. These barriers and strategies can be divided into three domains: “Knowledge and Expertise”, “Attention to Parental Needs” and “System and Organization”. Findings will be discussed below.

### Initial Concerns

Overall, parents reported first concerns about their child’s development at an average age of 22 months (with a range from 0 to 48 months), as is in line with previous research (Landa 2008; Zwaigenbaum et al., 2015). The nature of these initial concerns are behavioral problems and delays in language development, social communication and motor development of their child. Parents reported an average gap of 26 months between first concerns and receiving an ASD diagnosis for their child, which is also in line with previous studies (gaps varying between 1.5 and 3.5 years) (Crais et al., 2020; Crane et al., 2016; Zuckerman et al., 2015). A possible explanation for the gap between initial concerns and an ASD diagnosis given in the current study is that first line healthcare workers experience less severe concerns than parents, as experienced by the latter. This was also apparent in the focus group data, where parents felt that their concerns were not being acknowledged by a healthcare professional. Especially mothers experienced more severe initial concerns, which can explain why a majority of mothers signed up for participating in the focus group. Notably, parents reported a wide range of satisfaction ratings with the process between initial concerns and receiving an ASD diagnosis for their child with the majority of parents being content. In comparison, Crane et al. (2016) reported a dissatisfaction amongst parents regarding the diagnostic process. This dissatisfaction was partly the result of a long delay of 3.5 years between first contacting a healthcare professional and receiving a formal ASD diagnosis. In the current sample, though smaller, this delay was, on average, one year less and might be an explanation for the higher satisfaction score.

The ultimate goal of early identification is to improve and accelerate access to appropriate healthcare, not just speeding up the process of obtaining a diagnosis. However, a (working) diagnosis is often an important initial step for treatment (Blijd-Hoogewys et al., 2017; Gunnewijk & de Boer, 2021). Despite the relative positive satisfaction score, still plenty of improvement points arose amongst parents in the process of early identification which will be further discussed below.

### Barriers and Improvement Strategies Regarding Early Identification of ASD

The first barrier parents experienced in the early identification process of ASD was *limited knowledge and expertise* amongst first line healthcare professionals. Limited knowledge and expertise about ASD symptoms in infancy and toddlerhood in primary and preventive healthcare professionals is a commonly heard barrier, both mentioned by parents and professionals (Crais et al., 2020; Locke et al., 2020; Pinto-Martin et al., 2005; Snijder et al., 2021). Based on the frequency with which this barrier is reported, it can be considered a fundamental one to target in order to improve the early identification of ASD. To enhance the quality of care for children with ASD, the parents in the current sample strongly recommended additional training in the early behavioral symptoms of ASD to all primary and preventive care professionals. This proposed improvement strategy corresponds with the need for additional training as also previously expressed by PCPs and described in our parallel paper (Snijder et al., 2021), and is recommended in several other publications (Fenikilé et al., 2015; Mazurek et al., 2020, Oosterling et al., 2019).

The second barrier faced by parents in the early identification process of ASD emphasized the *importance of professionals attending to parental needs*. Several parents felt that their concerns were dismissed and marginalized by first line healthcare professionals. Unfortunately, this ‘wait-and-see’ approach is a well-known phenomenon. This approach where healthcare professionals show reassuring responses to initial parental concerns often result in a delayed referral for further assessment (Crais et al., 2020; Johnson et al., 2020; Oswald et al., 2015; Smith-Young et al., 2020; Zuckerman et al., 2015). The current study shows that a dismissive response often does not have a reassuring effect on parents, but instead seems to increase parents’ level of insecurity. Thus, instead of adapting a ‘wait-and-see’ approach, healthcare professionals should take parental concerns very seriously, monitor closely and refer to adequate care as quickly as possible when it comes to developmental concerns and/or suspicions of ASD. On the other hand, in clinical practice, there is another group of parents who don’t express first concerns regarding their child’s development, but where a healthcare professional is the first person to express concerns (Snijder et al., 2021). For this group of parents, an accelerated process in referring to adequate care might not be desirable and appropriate since parents need more time to process that their child’s development might be divergent. ASD in young children is complex and the timing and developmental course of early ASD symptoms vary amongst children (Zwaigenbaum et al., 2015). Still, it is highly recommended that first line healthcare workers let go of their “wait-and-see approach” and adopt a more family-centered care approach (Boshoff et al., 2018; Crais et al., 2020; Kuo et al., 2012; Locke et al., 2020). Family-centered care emphasizes the importance of a beneficial partnership between families and health care professionals with shared decision making as an important principle (Kuo et al., 2012; Locke et al., 2020). To establish shared decision making, an active role for parents during the decision making process is strongly encouraged (Crais et al., 2020). Examples on how to promote an active role for parents are the use of parent reports and specific screeners, such as the M-CHAT-RF (Modified-Checklist for Autism in Toddlers Revised and Follow Up; Robins et al., 2014) or the CoSoS (Communication and Social development Signs, previously known as ESAT; Dietz et al., 2006) or to let parents observe their child during daily routines, in order to reach consensus about developmental concerns and ease parent-professional conversations about the suspicions of ASD.

The last barrier that parents were challenged with in the process of early identification is related to the field of *System and Organization.* Parents often faced an inflexible and fragmented system with long waiting lists, that can be difficult to navigate. According to parents, this causes unnecessary delays and prolonged periods of stress. The complex infrastructure of the healthcare system and the stress it causes has also been reported in previous studies in other countries (Boshoff et al., 2018; Crais et al., 2020; Crane et al., 2016), which illustrates the importance of (a) parents receiving additional support in navigating the system and (b) a closer collaboration in regional networks between healthcare professionals that contributes to a less fragmented system. Parents in the current study expressed the need for customized care centered around families. They also mentioned the necessity of a case manager who helps them navigate the healthcare system and provides emotional support. The importance of a central contact or case manager can be viewed as a clinical best estimate (Blijd-Hoogewys et al., 2017) and an essential piece in improving early identification of children with or at risk of ASD. The lack of support parents experience in navigating the system again illustrates their need for a more family-centered care approach in the early identification process of ASD.

### Combining Parental and Professional Perspectives

In this last section, results of the current study will be integrated with the preventive care physicians (PCPs) viewpoints on the same topic (Snijder et al., 2021). In our previous study, PCPs expressed their concerns about having limited knowledge about early ASD symptoms, professional attitude in general towards early detection, problems in discussing initial concerns with parents, limited use of screening instruments, problems derived from cultural differences, and constraints regarding availability of healthcare services. Barriers related to these topics have a great overlap with the viewpoints of parents in the current study, with the exception of ‘the limited use of screening instruments’ and ‘problems derived from cultural differences barriers’. The absence of the screening and cultural barriers can be explained by the fact that parents are often not aware of the existence of specific screening instruments and that the current sample existed of native Dutch speaking participants only. Based on the overlapping barriers, parents and PCPs seem to experience the same issues regarding early identification of ASD, albeit from a different point of view.

**Fig. 2:**
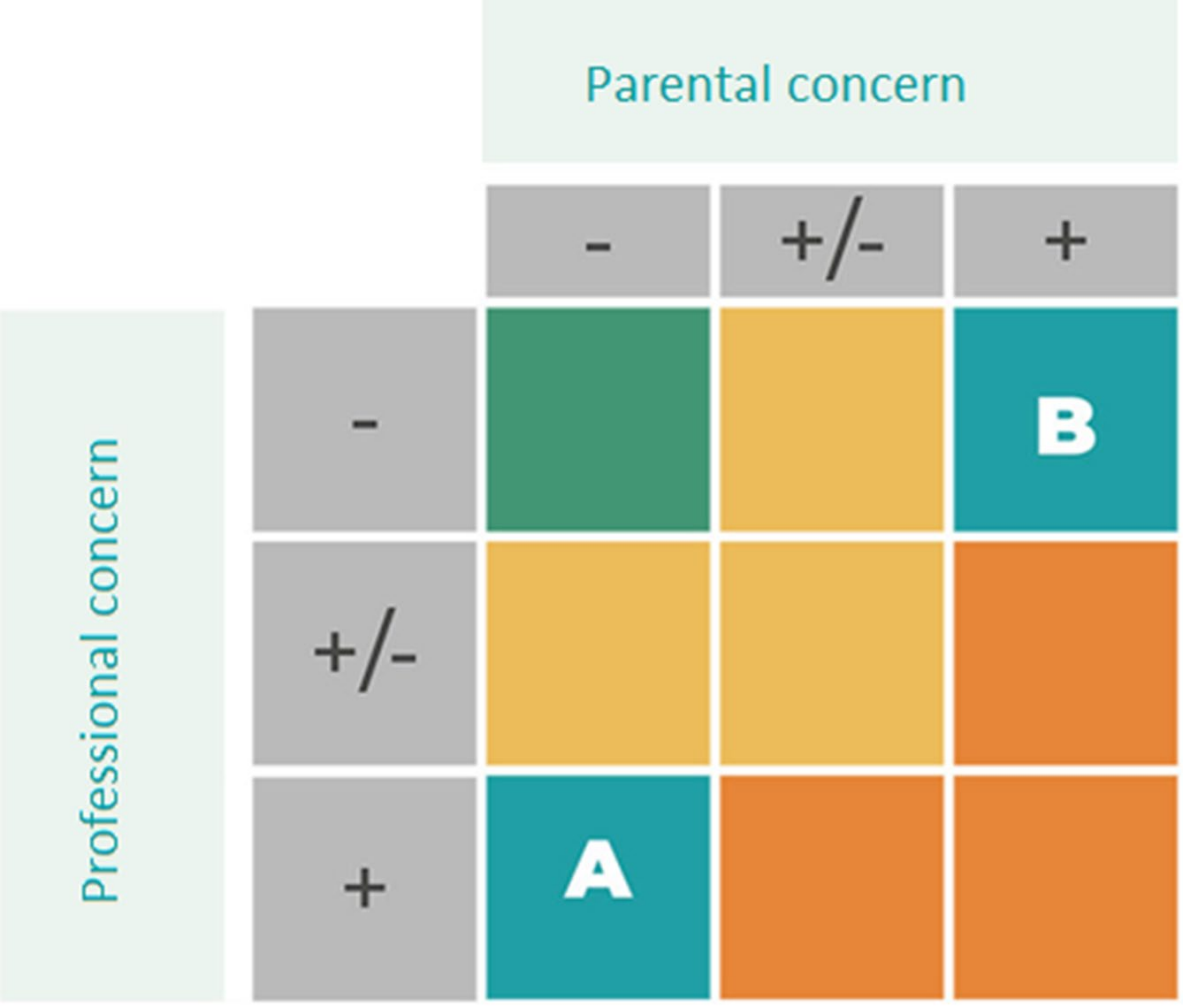
Parental concerns versus professional concerns, as developed by Oosterling et al., 2019

Given both perspectives, there seem to be two main difficulties in the parent-PCP conversations when there are concerns of the child being at risk for ASD. The first is where parents feel that their concerns are not acknowledged by PCPs at well-baby clinics and that they are told to ‘wait-and-see’. The second is where PCPs feel that parents find it difficult to recognize and accept that their child’s development might be divergent and PCPs therefore find it hard to discuss their suspicions of ASD and refer for diagnostic assessment. Amongst others, these two situations are visualized in Fig. 2 as first presented by Oosterling and colleagues (2019). The green compartment in the figure represents children where both parents and PCPs express no concerns and the child will probably develop typically. The red compartment represents children about whom both parents and PCPs have concerns and strongly suspect ASD. These “red” children represent a group that will generally be referred for diagnostic assessment at specialized mental healthcare centers at a very young age. The yellow compartment represents children where parents and PCPs have concerns, but the risk signals are less clear then in the “red” children. “Yellow” children might function on higher cognitive levels, have better language skills, or are maybe girls (with a less specific phenotype). This compartment represents the children who are often referred for diagnostic assessment at a later age. Finally, there are “A” and “B” children. The “A” group represents children where PCPs have concerns regarding the child’s development but parents have not, making it difficult for PCPs to motivate parents for diagnostic assessment (as described by Snijder et al., 2021). The “B” group represents the children described in the current paper, about whom parents experience concerns regarding their child’s development but are not acknowledged by PCPs. PCPs either do not recognize the early signs of ASD or PCPs think that the child is too young and that they should “wait-and-see” how the child will develop over time. Both groups are equally troublesome, since they contribute to a delay of approximately 2 years between initial concerns and diagnosis (Oosterling et al., 2019; Snijder et al., 2021).

The diagram may help in thinking about solutions for improving the early identification of ASD. As an example, to address problems related to both the ‘A’ and ‘B’ compartments, in the Netherlands, a free online training has been developed in order to improve professionals’ knowledge regarding ASD symptoms in infancy and toddlerhood (Oosterling & Dietz, 2017), along with an in-depth Live Online educational program that addresses early detection, ASD symptoms in infancy and toddlerhood, communicating with parents and referral (van’t Hof et al., 2020). Also, an easily accessible website has been developed by the national expert network autism in young children (www.autismejongekind.nl) in order to improve knowledge and raise awareness amongst professionals and parents. International examples of this are the “Learn the Signs: Act Early” initiative by the Centers for Disease Control and Prevention (www.firstsigns.org), and the Autism Navigator website (www.autismnavigator.com), an initiative by the Autism Institute at the Florida State University College of Medicine. All these strategies aim to increase awareness amongst both parents and professionals, acknowledging at the same time the importance of a shared decision making process in the early identification of children that would otherwise be stuck in the ‘B’ compartment, receiving adequate care later than preferred. An important note on increasing awareness of ASD symptoms in infancy and toddlerhood is that is has to go hand in hand with easily accessible and appropriate healthcare (Dietz et al., 2006; Oosterling et al., 2019; Snijder et al., 2021).

Therefore, a strategy to tackle the difficulties mainly of the ‘A’ compartment, but also possibly in the ‘B’ group, is offering accessible early interventions in a high-risk group. Without the need of an ASD diagnosis, these interventions are presumably more acceptable for those parents who do not yet have concerns (then for example a referral to a specialized center for infant psychiatry), and is at the same time an appropriate form of healthcare for the children in the ‘yellow’ compartment for whom referral for a diagnostic trajectory might not be applicable yet (because of unclear risk signals). Previous studies show promising outcomes of early interventions in at-risk groups and can reduce the severity of autism symptoms, enhance parent–child communication and improve parents’ responsiveness (Green et al., 2017; Kasari et al., 2014). Another new example of this is the BEAR intervention, recently developed in the Netherlands and based on effective naturalistic developmental behavioral early interventions (Bruinsma et al., 2020). BEAR stands for Blended E-health for children at eArly Risk and is an intervention offered to parents of young children identified as at risk for ASD. It concerns a short, parent adopted and easily accessible intervention that can be applied in the pre-diagnostic phase. For some children, this intervention will be the influx to more intensive and specialized healthcare, whereas for other children BEAR will function as a preventive intervention. BEAR is delivered by a skilled professional working in primary healthcare, ideally under supervision of a specialized mental healthcare expert. To this end, it also improves the collaboration between different healthcare organizations (an improvement strategy suggested by both parents and professionals). As such the training can also be perceived as a way of triage, where the professional supports parents in navigating the healthcare system, while parents themselves are actively involved during the whole process. Through all of this, it is hypothesized that easily accessible interventions such as BEAR will identify children that otherwise would be stuck in the ‘A’ and possibly ‘B’ compartment, and also provide adequate care to the ‘yellow’ children (Oosterling et al., 2019). Also, as known from the family-centered care principles, it is likely that the strategies of BEAR will enhance shared-decision making and boost collaboration between parents and professionals. Currently, the effectiveness of BEAR is being studied in a separate study.

### Limitations

The survey sample of the current study covered multiple regions of the Netherlands. However, not every region was represented (in both the online survey and the focus group) and the sample was limited to parents who agreed to participate. The focus group sample held a majority of high educated, Dutch native mothers. Although there was more diversity in the survey sample, participants were mostly Dutch natives. Overall, the survey sample was small with a high non-response rate, since no reminders could be sent to non-responders. Furthermore, parents were asked to participate when their child was aged 6 years or younger at the time of diagnosis and results might therefore not be representative for all parents with a child who has ASD. In the focus group, it seemed that only parents participated who had first concerns, whereas no parents were encountered where the professional expressed first concerns (as described in the PCP study). For both the survey and the focus group, parents were asked to reflect back on their experiences with the early identification process. Finally, a characteristic of qualitative research is that data analysis involves interpretation of the researchers, causing a risk of subjectivity. To limit this, the analytic process was done by multiple researchers with different educational backgrounds through extensive discussions regarding coding and developing themes.

## Conclusion and Clinical Implications

The current study shows that parents often have early initial concerns regarding their child development, but there is a risk that parents don’t feel that their concerns are being acknowledged by preventive and primary care professionals. In order to improve the early identification and initial care of children at risk of ASD, active investment in increasing knowledge and accessible access to suitable healthcare is recommended. Additionally, new strategies that focus on improving family-centered care and shared-decision making should be developed, evaluated and implemented. The proposed diagram can be a useful tool that will help to better understand how difficulties in early identification of ASD relate to perspectives of parents, professionals and child characteristics, and find ways to address difficulties from different perspectives.

## Supplementary Information

Below is the link to the electronic supplementary material.

Supplementary file1 (DOCX 15 kb)

Supplementary file2 (DOCX 21 kb)

Supplementary file3 (DOCX 21 kb)
