# Supplementary material for: Parental Experiences with Early Identification and Initial Care for their Child with Autism: Tailored Improvement Strategies
Source: J Autism Dev Disord. 2021 Sep 1;52(8):3473–85. doi: 10.1007/s10803-021-05226-y (PMC9296376; doi:10.1007/s10803-021-05226-y)
Supplement: Supplementary file 1 — Supplementary file1 (DOCX 15 kb) [file 10803_2021_5226_MOESM1_ESM.docx]

**Parental Experiences with Early Identification and Initial Care for their Child with Autism: Tailored Improvement Strategies.**

Journal of Autism and Developmental Disorders

Michelle I.J. Snijder^12^, Ilse P.C. Langerak^3^, Shireen P.T. Kaijadoe^1^, Marrit E. Buruma^4^, Rianne Verschuur^5^, Claudine Dietz^1^ Jan K. Buitelaar^12^ & Iris J. Oosterling^1^

^1^Karakter Child and Adolescent Psychiatry University Centre, Nijmegen, The Netherlands

^2^Department of Cognitive Neuroscience, Donders Institute for Brain, Cognition and Behaviour, Radboudumc, Nijmegen, The Netherlands

^3^ Apanta GGZ, Veldhoven, The Netherlands

^4^ INTER-PSY, Groningen, The Netherlands

^5^ Dr Leo Kannerhuis, Arnhem, The Netherlands

**Corresponding author**Correspondence concerning this article should be addressed to Michelle Snijder, Karakter Child and Adolescent Psychiatry University Centre, Reinier Postlaan 12, Nijmegen 6525 GC, The Netherlands
Email: [m.snijder@karakter.com](mailto:m.snijder@karakter.com)

**Supplementary Results***4. Experiences after obtaining diagnosis and treatment*
Parents additionally shared their experiences in obtaining an ASD diagnosis for their child and treatment. Since these experiences are important but are beyond the scope of the current study, these experiences are described in the Supplementary Results.

*4.1. Grieving process*
Whether the diagnosis comes as a shock or parents already expected it, the confirmation of it is often deeply painful and distressing for parents. Therefore, parents could not stress enough how important it is that healthcare professionals are aware and attend to parental grief resulting from receiving an ASD diagnosis for their child. Attendance to their grief can either occur through individual support or group support with other peers.

*4.2. Stigma*Some parents described their reticence of pursuing a diagnosis for their child at a young age. They fear that their child will be treated differently by others (grownups and peers) when the ASD diagnosis is confirmed. Additionally, some parents had concerns that their child might become angry with them or blame them at a later age for having pursued the ASD diagnosis.

*4.3 Treatment*Parents discussed the need for a “starters package” when their child had received an ASD diagnosis. According to parents, this “starters package” should provide practical tips about frequent behavioral problems in children with ASD (for example: some children with eating problems eat better of a plate with dividers so that food is separated) and provide information to navigate the complex roads of the healthcare system (for example: how to apply for additional financial support in their municipality).
 Furthermore, parents mentioned that they sometimes felt overwhelmed when numerous professionals were involved in the care and treatment of their child with ASD. Additionally, when new problems occurred after therapy ended, parents experienced difficulties in finding the right help and/or treatment. Parents wished for a better follow-up service after treatment in specialized mental healthcare centers had ended. They expressed that they might feel better, and possibly leave sooner, when it was possible to consult an ASD expert for quick questions, instead of going through the referral-waiting list-intake process once more.
