# Supplementary material for: Parental Experiences with Early Identification and Initial Care for their Child with Autism: Tailored Improvement Strategies
Source: J Autism Dev Disord. 2021 Sep 1;52(8):3473–85. doi: 10.1007/s10803-021-05226-y (PMC9296376; doi:10.1007/s10803-021-05226-y)
Supplement: Supplementary file 2 — Supplementary file2 (DOCX 21 kb) [file 10803_2021_5226_MOESM2_ESM.docx]

**Parental Experiences with Early Identification and Initial Care for their Child with Autism: Tailored Improvement Strategies.**

Journal of Autism and Developmental Disorders

Michelle I.J. Snijder^12^, Ilse P.C. Langerak^3^, Shireen P.T. Kaijadoe^1^, Marrit E. Buruma^4^, Rianne Verschuur^5^, Claudine Dietz^1^ Jan K. Buitelaar^12^ & Iris J. Oosterling^1^

^1^Karakter Child and Adolescent Psychiatry University Centre, Nijmegen, The Netherlands

^2^Department of Cognitive Neuroscience, Donders Institute for Brain, Cognition and Behaviour, Radboudumc, Nijmegen, The Netherlands

^3^ Apanta GGZ, Veldhoven, The Netherlands

^4^ INTER-PSY, Groningen, The Netherlands

^5^ Dr Leo Kannerhuis, Arnhem, The Netherlands

**Corresponding author**Correspondence concerning this article should be addressed to Michelle Snijder, Karakter Child and Adolescent Psychiatry University Centre, Reinier Postlaan 12, Nijmegen 6525 GC, The Netherlands
Email: [m.snijder@karakter.com](mailto:m.snijder@karakter.com)

**Supplementary table 1.** COREQ (COnsolidated criteria for REporting Qualitative research) Checklist for the study called “Parental Experiences with Early Identification and Initial Care for their Child with Autism. A mixed-method design.”

| **Topic** | | **Item No.** | **Guide Questions/Description** | **Reported on Page No.** |
| --- | --- | --- | --- | --- |
| **Domain 1: Research team and reflexivity** | |  |  |  |
| *Personal characteristics* | |  |  |  |
| Interviewer/facilitator | | 1 | Which author/s conducted the interview or focus group? | 7 |
| Credentials | | 2 | What were the researcher’s credentials? E.g. PhD, MD | 7, title page |
| Occupation | | 3 | What was their occupation at the time of the study? | 7 |
| Gender | | 4 | Was the researcher male or female? | 7 |
| Experience and training | | 5 | What experience or training did the researcher have? | 7 |
| *Relationship with participants* | |  |  |  |
| Relationship established | | 6 | Was a relationship established prior to study commencement? | 5 |
| Participant knowledge of the interviewer | | 7 | What did the participants know about the researcher? E.g. personal goals, reasons for doing the research | 5 |
| Interviewer characteristics | | 8 | What characteristics were reported about the interviewer/ facilitator? E.g. Bias, assumptions, reasons and interests in the research topic | 7 |
| **Domain 2: Study design** | |  |  |  |
| *Theoretical framework* | |  |  |  |
| Methodological orientation and Theory | | 9 | What methodological orientation was stated to underpin the study? E.g. grounded theory, discourse analysis, ethnography, phenomenology, content analysis | 8 |
| *Participant selection* | |  |  |  |
| Sampling | | 10 | How were participants selected? E.g. purposive, convenience, consecutive, snowball | 5 |
| Method of approach | | 11 | How were participants approached? E.g. face-to-face, telephone, mail, email | 5 |
| Sample size | | 12 | How many participants were in the study? | 5 |
| Non-participation | | 13 | How many people refused to participate or dropped out? Reasons? | 5 |
| *Setting* | |  |  |  |
| Setting of data collection | | 14 | Where was the data collected? E.g. home, clinic, workplace | 7 |
| Presence of non-participants | | 15 | Was anyone else present besides the participants and researchers? | 7 |
| Description of sample | | 16 | What are the important characteristics of the sample? E.g. demographic data, date | 6 |
| *Data collection* | |  |  |  |
| Interview guide | | 17 | Were questions, prompts, guides provided by the authors? Was it pilot tested? | 7 |
| Repeat interviews | | 18 | Were repeat inter views carried out? If yes, how many? | 5, 7, 8 |
| Audio/visual recording | | 19 | Did the research use audio or visual recording to collect the data? | 8 |
| Field notes | | 20 | Were field notes made during and/or after the interview or focus group? | 7,8 |
| Duration | | 21 | What was the duration of the inter views or focus group? | 7 |
| Data saturation | | 22 | Was data saturation discussed? | 8 |
| Transcripts returned | | 23 | Were transcripts returned to participants for comment and/or correction? | 8 |
| **Domain 3: analysis and findings** |  | |  |  |
| *Data analysis* |  | |  |  |
| Number of data coders | 24 | | How many data coders coded the data? | 8 |
| Description of the coding tree | 25 | | Did authors provide a description of the coding tree? | N/A |
| Derivation of themes | 26 | | Were themes identified in advance or derived from the data? | 8 |
| Software | 27 | | What software, if applicable, was used to manage the data? | 8 |
| Participant checking | 28 | | Did participants provide feedback on the findings? | 8 |
| *Reporting* |  | |  |  |
| Quotations presented | 29 | | Were participant quotations presented to illustrate the themes/findings? Was each quotation identified? E.g. participant number | Table 2 |
| Data and findings consistent | 30 | | Was there consistency between the data presented and the findings? | 10,11,12,13 Table 2 |
| Clarity of major themes | 31 | | Were major themes clearly presented in the findings? | 10,11,12,13 |
| Clarity of minor themes | 32 | | Is there a description of diverse cases or discussion of minor themes? | 10,11,12,13 |

*Note.* Developed from: Tong A, Sainsbury P, Craig J. Consolidated criteria for reporting qualitative research (COREQ): a 32-item checklist for interviews and focus groups. *International Journal for Quality in Health Care*. 2007. Volume 19, Number 6: pp. 349 – 357
