# Supplementary material for: Parental Experiences with Early Identification and Initial Care for their Child with Autism: Tailored Improvement Strategies
Source: J Autism Dev Disord. 2021 Sep 1;52(8):3473–85. doi: 10.1007/s10803-021-05226-y (PMC9296376; doi:10.1007/s10803-021-05226-y)
Supplement: Supplementary file 3 — Supplementary file3 (DOCX 21 kb) [file 10803_2021_5226_MOESM3_ESM.docx]

**Parental Experiences with Early Identification and Initial Care for their Child with Autism: Tailored Improvement Strategies.**

Journal of Autism and Developmental Disorders

Michelle I.J. Snijder^12^, Ilse P.C. Langerak^3^, Shireen P.T. Kaijadoe^1^, Marrit E. Buruma^4^, Rianne Verschuur^5^, Claudine Dietz^1^ Jan K. Buitelaar^12^ & Iris J. Oosterling^1^

^1^Karakter Child and Adolescent Psychiatry University Centre, Nijmegen, The Netherlands

^2^Department of Cognitive Neuroscience, Donders Institute for Brain, Cognition and Behaviour, Radboudumc, Nijmegen, The Netherlands

^3^ Apanta GGZ, Veldhoven, The Netherlands

^4^ INTER-PSY, Groningen, The Netherlands

^5^ Dr Leo Kannerhuis, Arnhem, The Netherlands

**Corresponding author**Correspondence concerning this article should be addressed to Michelle Snijder, Karakter Child and Adolescent Psychiatry University Centre, Reinier Postlaan 12, Nijmegen 6525 GC, The Netherlands
Email: [m.snijder@karakter.com](mailto:m.snijder@karakter.com)

**Survey “Experiences with healthcare”**

Demographics

In this section, some background information will be asked about your child and your family.

When completing the questions, keep in mind your child with a diagnosis in the autism spectrum and who was 6 years old or younger at the time of diagnosis. Are there several children in the family who meet these criteria? Then we would like to ask you to keep the oldest child in mind.

For most questions, all you have to do is choose the correct answer and select the appropriate box. If you are unsure between two answer options, we ask you to make a choice. It is better for the research if all the data are entered and you choose the answer that best applies.

1. Who completes this questionnaire?

o Biological mother

o Biological father

o Stepmother

o Stepfather

o Adopting mother

o Adoptive father

o Foster mother

o Foster father

o Other, namely: _______________

2. What is the sex of your child?

o Boy
o Girl

3. What is your child's age? Please indicate the age of your child in years and months (for example: 4 years and 7 months)

4. What nationality has your child?

o Dutch

o Other, namely: ________________

5. In which country was your child born?

o The Netherlands

o Suriname

o Netherlands Antilles

o Aruba

o Morocco

o Turkey

o Germany

o Other, namely: ________________

6. In which province does your child live?

o Drenthe

o Flevoland

o Friesland

o Gelderland

o Groningen

o Limburg

o Noord Brabant

o Noord Holland

o Overijssel

o Utrecht

o Zeeland

o Zuid Holland

7. Does your child have (half) brothers and / or (half) sisters?

o Yes 🡪 Continue with the questions below

o No 🡪 Continue to General questions about biological father

8. For every (half)brother and/or (half)sister:

8a) How old is this (half) brother / (half) sister? Please indicate the age in years and months.

________________________

8b) Does one of the following problems occur with this (half) brother / (half) sister? Multiple answers are correct

o None of the following

o ADHD

o Aggression problems

o Anxiety problems

o Autism spectrum disorder

o Depressed, gloomy

o Compulsive thoughts and / or actions

o Eating problems

o Behavioral disorder

o Learning disability

o Mental disability

o Tics

o Trauma

o Self-injury

o I don't know

o Other, namely__________________________

9.What is the age of the biological father of the participating child? Please indicate the age in years and months.

10.In which country was the biological father born?

o The Netherlands

o Suriname

o Netherlands Antilles

o Aruba

o Morocco

o Turkey

o Germany

o I don't know

o Other, namely: ________________

11. What language does the biological father speak with the participating child?

o Dutch

o Not applicable (e.g. there is no contact with the biological father)

o Other ___________________

12. What is the highest education the biological father has completed?

o No education: did not complete primary school

o Primary education: primary school, special education

o Junior general secondary

o Senior secondary vocational|

o Pre-university

o University

o Higher professional

13. Does the biological father have one or more of the following psychological / psychiatric problems / complaints? Or did the biological father have these? Multiple answers are correct.

o None of the following

o ADHD

o Aggression problems

o Anxiety problems

o Autism spectrum disorder

o Bipolar disorder

o Borderline personality disorder

o Depressed, gloomy

o Compulsive thoughts and / or actions

o Eating problems

o Behavioral disorder

o Learning disability

o Mental disability

o Psychosis, schizophrenia

o Stress / burnout / overworked

o Tics

o Trauma

o Addiction problems

o Suicidal thoughts, attempt

o I don't know

o Other ___________________

14.What is the age of the biological mother of the participating child? Please indicate the age in years and months.

15. In which country was the biological mother born?

o The Netherlands

o Suriname

o Netherlands Antilles

o Aruba

o Morocco

o Turkey

o Germany

o I don't know

o Other, namely: ________________

16. What language does the biological mother speak with the participating child?

o Dutch

o Not applicable (e.g. there is no contact with the biological mother)

o Other ___________________

17. What is the highest education the biological mother has completed?
o No education: did not complete primary school

o Primary education: primary school, special education

o Junior general secondary

o Senior secondary vocational|

o Pre-university

o University

o Higher professional

18. Does the biological mother have one or more of the following psychological / psychiatric problems / complaints? Or did the biological mother have these? Multiple answers are correct.

o None of the following

o ADHD

o Aggression problems

o Anxiety problems

o Autism spectrum disorder

o Bipolar disorder

o Borderline personality disorder

o Depressed, gloomy

o Compulsive thoughts and / or actions

o Eating problems

o Behavioral disorder

o Learning disability

o Mental disability

o Psychosis, schizophrenia

o Stress / burnout / overworked

o Tics

o Trauma

o Addiction problems

o Suicidal thoughts, attempt

o I don't know

o Other ___________________

**First concerns**

1. How old was your child when first concerns about his / her development arose? Try to be as accurate as possible. Enter the exact age, in years and months (for example 3 years and 4 months old)

2. What were primary concerns? Multiple answers can be given.

o Language development (e.g. no babbling, no use of short sentences, previously spoken words that disappeared, etc.)

o Motor development (e.g. delayed withwalking and / or crawling, moving awkwardly/clumsy, difficulty with fine motor skills such as holding a pencil, etc.)

o Social contact and communication (e.g. difficulty having fun together, not smiling at others, not waving, etc.)

o Play development (e.g. not playing, constantly doing the same with toys, little imagination, always organizing / lining up things, etc.)

o Behavioral problems (e.g. anger attacks and / or tantrums, anxious behavior, self-determining behavior, etc.)

o Medical problems (e.g. epilepsy, metabolic disease, etc.)

o Toilet training problems

3. Can you indicate the severity of the first concerns?

a) for mother

Few concerns Severe concerns

1 2 3 4 5

o not applicable

b) for father

Few concerns Severe concerns

1 2 3 4 5

o not applicable

c) for the general practitioner

Few concerns Severe concerns

1 2 3 4 5

o not applicable

d) preventive care physician

Few concerns Severe concerns

1 2 3 4 5

o not applicable

e) Other healthcare professionals

Few concerns Severe concerns

1 2 3 4 5

o not applicable

4. Which professional referred your child for further help?

o Preventive care physicians / preventive nurse

o General practitioner

o Other, namely _______________

**In search for help**

Before a child receives an ASD (autism spectrum disorder) diagnosis, a whole process often precedes it, in which various care providers or healthcare centers are visited. How many different care providers / healthcare centers did you and your child see / visit before your child was diagnosed with ASD? By different care providers we mean, for example, a speech therapist, psychologist, pediatrician, physiotherapist, etc. The general practitioner and youth doctor of the health clinic are not covered by this. So count the number of care providers / assistance agencies before the diagnosis was made.

1. How many different care providers did you and your child visit before your child was diagnosed with ASD?

o 0

o 1

o 2

o 3

o 4

o 5

o More than 5

2. Which care providers were these?

**Diagnosis**

1. What was your child diagnosed with?

o Autistic disorder

o PDD-NOS

o Asperger's syndrome

o Autism spectrum disorder

o Characteristics of an autism spectrum disorder

o Other: ________________________________

2. How old was your child when he / she was diagnosed? Try to be as accurate as possible. Enter the exact age, in years and months
_______________________________

3. Has your child received any other diagnosis in addition to the above diagnosis?

o Yes 🡪 Go to question 4

o No 🡪 Go to question 5

4. What other diagnosis has your child received, in addition to the aforementioned diagnosis? Multiple answers can be given

o ADHD

o Learning disability

o Behavioral disorder

o Mental disability

o Anxiety disorder

o Other__________________

5. Looking back on the entire process (from initial care to making the diagnosis, completed in question 1), how satisfied are you with the care provided during the process?

Not at all. Very much

1 2 3 4 5

Space for comments: _________________________________________________________________________________________________________________________________________________________________________________________________________________________________

**Treatment after diagnosis**

There are a number of therapies and / or treatments that children can receive after an ASD diagnosis has been established.

We would like you to answer which treatments / therapies your child has received or is currently receiving in the following questions?

It concerns the following treatments:

- Social skills training

- Pivotal response treatment (PRT)

- Floorplay

- Discrete trial teaching (DTT) or applied behavior analysis (ABA)

- JASPER training

- Music therapy

- Play therapy

- Medication
- Other

1. Social skills training

1. My child has received social skills training

o Yes 🡪 Go to question 1A

o No 🡪 Go to question about PRT

1a. How old was your child when he / she started social skills training? Enter the exact age in years and months.

____________________________________________

1b. Looking back, how satisfied are you with this treatment?

Not at all. Very much

1 2 3 4 5

2. My child has received PRT (pivotal response treatment).

o Yes 🡪 Go to question 2a

o No 🡪 Go to question about Floorplay

2a. How old was your child when he / she started PRT? Enter the exact age in years and months

____________________________________________

2b. Looking back, how satisfied are you with this treatment?

Not at all. Very much

1 2 3 4 5

3. My child has received Floorplay

o Yes 🡪 Go to question 3a

o No 🡪 Go to question about DTT/ABA

3a. How old was your child when he / she started Floorplay? Enter the exact age in years and months

____________________________________________

3b. Looking back, how satisfied are you with this treatment?

Not at all. Very much

1 2 3 4 5

4. My child has received DTT (discrete trial teatching) or ABA (applied behaviour analysis)

o Yes 🡪 Go to question 4a

o No 🡪 Go to question about JASPER training

4a. How old was your child when he / she started DTT or ABA? Enter the exact age in years and months

____________________________________________

4b. Looking back, how satisfied are you with this treatment?

Not at all. Very much

1 2 3 4 5

5. My child has received JASPER training

o Yes 🡪 Go to question 5a

o No 🡪 Go to question about music therapy

5a. How old was your child when he / she started JASPER training? Enter the exact age in years and months

____________________________________________

5b. Looking back, how satisfied are you with this treatment?

Not at all. Very much

1 2 3 4 5

6. My child has received music therapy

o Yes 🡪 Go to question 6a

o No 🡪 Go to question about play therapy

6a. How old was your child when he / she started JASPER training? Enter the exact age in years and months

____________________________________________

6b. Looking back, how satisfied are you with this treatment?

Not at all. Very much

1 2 3 4 5

7. My child has received play therapy

o Yes 🡪 Go to question 7a

o No 🡪 Go to question about “other”

7a. How old was your child when he / she started JASPER training? Enter the exact age in years and months

____________________________________________

7b. Looking back, how satisfied are you with this treatment?

Not at all. Very much

1 2 3 4 5

8. Did you child received any other treatments?

o Yes 🡪 go to question 8a
o No

8a. Which other treatments did your child receive?
Treatment 1:_________
Treatment 2:_________
Treatment 3:_________

8b. How old was your child when he/she started treatment?
Treatment 1:_________
Treatment 2:_________
Treatment 3:_________

8c. Looking back, how satisfied are you with this treatment?

Treatment 1:

Not at all. Very much

1 2 3 4 5

Treatment 2:

Not at all. Very much

1 2 3 4 5

Treatment 3:

Not at all. Very much

1 2 3 4 5

Medication

1. Has your child been on medication? Or does your child receive medication?

o Yes 🡪 Go to question 2

o No 🡪 Go to question about parental guidance

2. What medication is your child using?
______________________________

**Parent therapy**

There are a number of therapies or counseling contacts that parents can get after ASD has been diagnosed in their child. Can you indicate per therapy or counseling contact whether you received it?

This concerns the following therapies / counseling contacts:

- Parent group with explanation about ASD

- Parental guidance

- Ambulatory home guidance

- Videohome training

- Family therapy

- Other therapies or counseling contacts

1. Have you yourself had therapy or counseling contacts that were related to the upbringing and support of your child?

o Yes 🡪 Go to question 2

o No 🡪 Go to question about education

2. Did you participate in a parent group in which an explanation about ASD was given?

o Yes 🡪 Go to question 2a.

o No 🡪 Go to question about parental guidance

2a Looking back, how satisfied are you with this treatment?

Not at all. Very much

1 2 3 4 5

3. Have you received parental guidance?
Explanation: parental guidance consists of conversations that you often have 1 to 2 times a week with a psychologist specialized in ASD.

o Yes 🡪 Go to question 3a.

o No 🡪 Go to the question about ambulante thuisbegeleiding

3a Looking back, how satisfied are you with this treatment?

Not at all. Very much

1 2 3 4 5

4. Have you received ambulante thuisbegeleiding?

o Yes 🡪 Go to question 4a.

o No 🡪 Go to the question about video home training

4a Looking back, how satisfied are you with this treatment?

Not at all. Very much

1 2 3 4 5

5. Have you received vide home training?

o Yes 🡪 Go to question 5a.

o No 🡪 Go to the question about family therapy

5a Looking back, how satisfied are you with this treatment?

Not at all. Very much

1 2 3 4 5

6. Have you received family therapy?

o Yes 🡪 Go to question 6a.

o No 🡪 Go to the question about other treatments

6a Looking back, how satisfied are you with this treatment?

Not at all. Very much

1 2 3 4 5

7. Have you received other treatments?

o Yes 🡪 Go to question 7a
o No 🡪 Go to the question about education

7a. Which treatments did you receive?

Treatment 1 ___________________
Treatment 2___________________
Treatment 3___________________

7b. Looking back, how satisfied are you with this treatment?

Treatment 1:

Not at all. Very much

1 2 3 4 5

Treatment 2:

Not at all. Very much

1 2 3 4 5

Treatment 3:

Not at all. Very much

1 2 3 4 5

**Education**

1. What kind of education does your child receive?

o Regular daycare

o Special daycare

o Regular (primary) education

o Special (primary) education

o My child does not go to a daycare / school.

o Other, namely;_____________________

**Concluding questions**

1. Overall (the entire process from first concerns to treatment), to what extent has the care you or your child received met your wishes?

Not at all. Very much

1 2 3 4 5

2. Looking back on the entire process, what are you mainly satisfied with?
_________________________________________________________________________________________________________________________________________________________________________________________________________________________________

3. Looking back on the entire process, what would you like to see improved?
_________________________________________________________________________________________________________________________________________________________________________________________________________________________________
